# Monocyte Subsets in Atherosclerosis and Modification with Exercise in Humans

**DOI:** 10.3390/antiox7120196

**Published:** 2018-12-19

**Authors:** Ning Hong Aw, Elisa Canetti, Katsuhiko Suzuki, Jorming Goh

**Affiliations:** 1 School of Chemical and Life Sciences, Singapore Polytechnic, Singapore 139651, Singapore; nhong002@gmail.com; 2 Faculty of Health Sciences and Medicine, Bond University, Robina QLD 4226, Australia; ecanetti@bond.edu.au; 3 Faculty of Sport Sciences, Waseda University, Tokorozawa 359-1192, Japan; katsu.suzu@waseda.jp; 4 Ageing Research Institute for Society & Education (ARISE), Nanyang Technological University, Singapore 639798, Singapore; 5 Exercise Medicine & Physiology Laboratory, Lee Kong Chian School of Medicine, Nanyang Technological University, Singapore 639798, Singapore

**Keywords:** monocytes, exercise training, atherosclerosis, inflammation

## Abstract

Atherosclerosis is a progressive pathological remodeling of the arteries and one of its hallmarks is the presence of chronic inflammation. Notably, there is an increased proportion and activation state of specific monocyte subsets in systemic blood circulation. Monocyte subsets have distinct contributions to the formation, progression, and destabilization of the atherosclerotic plaque. Strong clinical and epidemiological studies show that regular aerobic exercise mitigates the progression of cardiovascular disease. In fact, aerobic fitness is a powerful predictor of cardiovascular mortality in adults, independent of traditional risk factors such as hypertension and hyperlipidemia. Acute bouts and chronic exercise training modulate monocyte behavior, ranging from their recruitment from the bone marrow or marginal pool, to tissue margination and functional changes in cytokine and chemokine production. Such modulation could reflect a potential mechanism for the cardio-protective effect of exercise on atherosclerosis. This review summarizes the current knowledge of monocyte subsets and highlights what is known about their responses to exercise.

## 1. Introduction

Monocytes are immune cells originating from myeloid precursors and modulate the host inflammatory response. Monocytes provide non-specific immune surveillance, wound healing, and tissue remodeling. Three different sub-populations have been identified in humans and classified primarily on the expression of clusters of differentiation (CD)14 and CD16 on the cell surface. They comprise: (i) classical monocytes (CD14^++^CD16^−^), (ii) intermediate monocytes (CD14^++^CD16^+^), and (iii) non-classical monocytes (CD14^+^CD16^++^) [1]. These monocyte subsets differ in gene expression and cytokine production, antigen processing and presentation, as well as the capacity for inducing angiogenesis [2] and the propensity to differentiate into dendritic cells or activate T-lymphocytes [3].

More recently, there has been increased inquiry into the role of monocyte subsets in modulating cardiovascular disease risk. Research on pre-clinical animal models and human patients with atherosclerosis demonstrated the accumulation of monocytes in atherosclerotic plaques, which supported the concept that the immune system participates in the pathogenesis of coronary artery disease [4].

The cardioprotective effects of regular exercise on reducing the risks of, and progression of cardiovascular disease are well known. Regular exercise training has been documented to improve vascular compliance [5,6] and endothelial function [7]. Such benefits associated with changes in the architecture of the vasculature may involve changes in the phenotypes and functions of circulating immune cells, such as monocytes. We will provide an overview of the presently known subsets of monocytes in humans, their roles in the progression of atherosclerosis and how regular exercise training may modify their frequency and function in circulation. 

While narrative, this review has taken a structured approach. We performed a search of well-established databases such as Cochrane, EBSCO, Medline via Ovid, PubMed, and Scopus. The search terms (including their derivatives, i.e., plural) were monocyte, monocyte subset, CD14, CD16, exercise, atherosclerosis, atherosclerosis plaque, cardiovascular disease, coronary artery disease, and vascular epithelium. Our search included all fields and MeSH terms. Articles found in this search were selected based on relevance to the narrative and recency. Further, hand-picked articles were also included.

## 2. Monocytes Contribute to the Pathogenesis of Atherosclerosis 

Cardiovascular disease (CVD) is the primary cause of mortality and morbidity worldwide, with a 41% increase in mortality reported between 1990 and 2013 [8]. A primary contributor to CVD mortality is atherosclerosis, a progressive vessel disease characterized by the initial accumulation of lipid and fibrous elements on the arterial lumen [9]. Atherosclerosis is usually triggered by a hypoxic environment, which results in endothelial dysfunction [10], tissue inflammation, and the recruitment of circulating leukocytes via integrin-specific receptors [11,12,13]. Broadly speaking, pro-inflammatory mediators of the immune system act on dysfunctional endothelium surfaces of the arteries and their interactions with modified (oxidized) lipoproteins exacerbate the inflammatory process [10]. This inflammatory response then gives rise to the formation of advanced lesions that exudate into the arterial lumen. Complex lesions can occlude the arteries and reduce blood flow and oxygen to the myocardium. At the same time, such lesions are susceptible to rupture, which release intracellular contents, such as tissue factors, into the lumen and initiate a cascade of events leading to coagulation, platelet recruitment, and eventually the formation of thrombi [10]. Thrombosis has been attributed as a cause of acute cardiac events, such as unstable angina, myocardial infarction, or stroke.

Monocytes infiltrate into the sub-endothelial space upon binding to their integrin receptors with the adhesion molecules on the endothelium surface. The first indication of atherosclerotic change is usually thickening of the *tunica intima* of the artery [14]. Monocytes differentiate into tissue macrophages, and in the context of atherosclerosis, up to 60% of cells in the necrotic core of atheromatous plaques were found to express the Leu-M3 antigen [15], which is indicative of a macrophage phenotype. Monocytes induce mitotic activity of the endothelial and smooth muscle cells via the production of a platelet-derived growth factor [16]. Plaque monocytes/macrophages will then ingest oxidized low-density lipoprotein within the intima of the atherosclerotic lesion and become “foam cells.” These “foam cells” subsequently stimulate smooth muscle cell migration, eventually resulting in vascular remodeling [17]. Plaque monocytes/macrophages contribute to the thrombotic complications of atherosclerosis. These leukocytes supply the majority of the enzymes that catabolize collagen, which is the main component of the plaque’s fibrous cap [4]. Excessive production of the interstitial collagenase members of the matrix metalloproteinase family, which are involved in the remodeling of the arterial extracellular matrix, may induce destabilization of atherosclerotic plaques. This would eventually result in rupture of the atherosclerotic plaque and partial or complete thrombosis of the coronary artery. 

## 3. Monocyte Subsets

### 3.1. Classical Monocytes (CD14^++^CD16^−^)

CD14^++^CD16^−^ monocytes make up the largest population of the monocyte subsets and constitute about 85–90% of the total monocyte population in healthy individuals [1]. This subset expresses high levels of chemokine receptor type 2 (CCR2), a receptor for monocyte chemotactic protein (MCP)-1, and low or undetectable levels of CX_3_C chemokine receptor 1 (CX_3_CR1) [18]. CD14 binds lipopolysaccharide (LPS)-binding proteins with Toll-like receptor (TLR) 4, thus functioning as a co-receptor for bacterial LPS [19,20]. As a pattern recognition receptor (PRR), CD14 can also detect other pathogen-associated molecular patterns (PAMPs) such as lipopeptides [21] and free fatty acids [22]. CD16 is a fragment crystallizable region (Fc)-γ receptor III and facilitates antibody-dependent cellular cytotoxicity (ADCC) by binding to the Fc portion of various antibodies [23]. CD14^++^CD16^−^ monocytes also produce high amounts of the anti-inflammatory cytokine, interleukin (IL)-10, rather than tumor necrosis factor (TNF)-α and IL-1β, in response to LPS in vitro [24,25,26]. The PRRs, such as TLR2 and TLR4, are important in the pathogenesis of atherosclerosis, because of their interaction with oxidized lipids to drive the inflammatory responses [27]. Furthermore, oxidized lipids can down-regulate the production of IL-10 in monocytes and lead to a shift to a pro-inflammatory state [28].

### 3.2. Non-Classical Monocytes (CD14^+^CD16^++^)

CD14^+^CD16^++^ monocytes make up about 10% of the total monocyte population [29] and express an abundance of CX_3_CR1 [18] but not CCR2 [30]. High expression of CX_3_CR1 in non-classical monocytes is associated with their migration to inflamed endothelia [18]. CD14^+^CD16^++^ monocytes are potent producers of pro-inflammatory cytokines, such as IL-1β, TNF-α, and IL-6 [31], and have also been described as “pro-inflammatory” monocytes. They also adhere faster to activated endothelium than CD14^++^CD16^−^ monocytes [18] and are effective antigen presenters [32,33]. Other studies [34,35] demonstrated that this subset of monocytes consists of two sub-populations with different activities: CD14^+^CD16^+^ and CD14^dim^CD16^+^. CD14^+^CD16^+^ monocytes possess phagocytic activity and are the main producers of pro-inflammatory cytokines (TNF-α, IL-1β) in response to LPS. Conversely, CD14^dim^CD16^+^ monocytes have poor phagocytic activity and are low producers of TNF-α and IL-1β in response to LPS. 

### 3.3. Intermediate Monocytes (CD14^++^CD16^+^)

Prior to 2003, researchers reported only CD14^++^CD16^−^ and CD14^+^CD16^++^ monocytes and did not further distinguish the CD14^++^CD16^+^ subset. Some have suggested that this population is a transient subset that can mature into CD14^++^CD16^−^ monocytes [36]. The CD14^++^CD16^+^ monocyte subset comprises approximately 5% of total monocytes and is a significant producer of TNF-α and IL-10 in response to LPS [26,37]. 

Further research has demonstrated characteristics that clearly distinguish the CD14^++^CD16^+^ monocyte subset from the CD14^++^CD16^−^ and CD14^+^CD16^++^ subsets. Wong et al. [25] have identified increased expression of major histocompatibility complex (MHC)II, GDNF family receptor (GFR)α2, and C-type lectin domain family (CLEC)10A in CD14^++^CD16^+^ monocytes when compared to both CD14^++^CD16^−^ and CD14^+^CD16^++^ populations. Through gene ontology analysis, Zawada et al. [38] characterized distinct immune capabilities of the CD14^++^CD16^+^ subset such as antigen processing and presentation, inflammation, and monocyte activation and angiogenesis. CD14^++^CD16^+^ monocytes also have upregulated pro-angiogenic markers including endoglin (ENG), Tie-2, and kinase insert domain receptor (KDR). Compared with CD14^++^CD16^−^ monocytes, CD14^++^CD16^+^ monocytes have greater capacity for trans-endothelial migration, phagocytosis and reactive oxygen species (ROS) generation [38].

**Figure 1:**
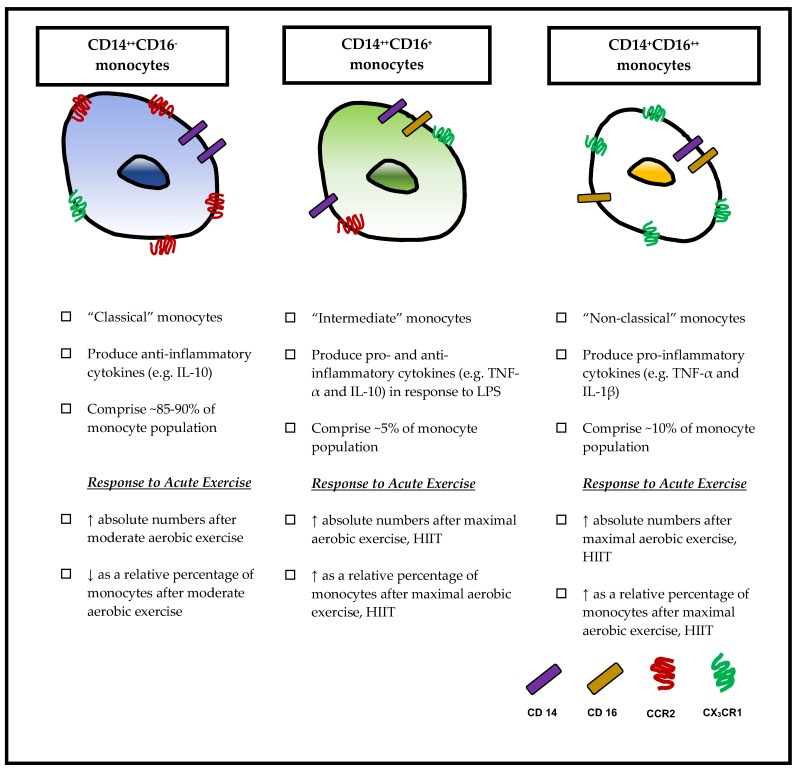
Monocyte subsets and responses to acute exercise.

Taken together, we have discussed how monocyte subsets possess unique characteristics that illustrate their different roles in the pathogenesis of atherosclerosis (Figure 1). Recent experimental studies have also shown that monocyte subsets do not exist discretely, but rather, they belong to a dynamic continuum. An elegant kinetic study using deuterium labeling in vivo with human volunteers has shown that monocytes undergo a transitory state during experimentally-induced endotoxemia [36]. In this study, CD14^++^CD16^−^ monocytes were the first subtype to reappear in circulation 4 h after endotoxemia-induced acute monocytopenia. After 24 h, this subset had transitioned into CD14^++^CD16^+^ and CD14^+^CD16^++^ monocytes and by the seventh day, the monocyte subsets returned to steady state, suggesting that the resolution of inflammation coincided with the clearance of the monocyte subsets in circulation.

## 4. Role of Monocyte Subsets in Atherosclerosis

Research in the last decade has seen an increase in the number of cross-sectional and cohort studies that investigated the contribution of monocyte subsets to atherosclerosis. In the Malmo Diet and Cancer study, which is a long-term Swedish epidemiological study spanning almost eight decades, 6103 participants from the original birth cohort participated in the study’s sub-arm of carotid artery disease [29]. Participants with the highest tertile of CD14^++^CD16^−^ monocytes had a hazard ratio of 1.66 (CI: 1.02–2.72) for developing incident CVD, independent of traditional risk factors, such as gender, age, or HDL cholesterol, compared with participants in the lowest tertile. Although baseline CD14^++^CD16^−^ monocyte subsets predicted CVD risk in this cohort, there was no association with mean intima-media thickness (IMT) of the common carotid artery. However, the percentage of CD16^+^ monocytes was negatively associated with IMT. 

In contrast to findings from the Malmo Diet and Cancer study, others have reported CD14^++^CD16^+^ monocyte subsets to have a greater contribution in the pathogenesis of atherosclerosis. In the HOM SWEET HOMe (Heterogeneity of Monocytes in Subjects Who Undergo Elective Coronary Angiography—The Homburg Evaluation) study, 951 patients who were referred for elective coronary angiography donated blood specimens with monocytes harvested for flow cytometry, and were tracked prospectively for about 2.5 years [39]. Patients were categorized into quartiles of total and subsets of monocyte cell counts, and COX regression analysis adjusted for age and sex demonstrated that, compared with patients in the lowest quartile of CD14^++^CD16^+^ monocytes, those in the highest quartile had the largest hazard ratios (HR: 3.899, CI: 1.86–8.17) for developing an adverse cardiovascular event, such as cardiovascular death, myocardial infarction, or non-hemorrhagic stroke. In 588 asymptomatic middle-aged adults, there was a strong association between CD14^++^CD16^+^ monocyte subset counts and calcified plaque scores as determined using computer tomography. Such a finding suggests that CD14^++^CD16^+^ monocytes may play a more important role than the other two subsets in the progression of coronary stenosis. 

The discrepancy in the two cohort studies may be explained by methodological differences, wherein monocyte subsets were enumerated from frozen whole blood samples in the Malmo Diet and Cancer study, unlike the HOM SWEET HOMe study, where fresh blood samples were used. Hence, the distribution and phenotype of the monocyte subsets could have been changed by long-term storage, as opposed to freshly isolated samples. Furthermore, the timing of blood processing also influences the expression of CD16 on these monocyte subsets. When sample processing was delayed by 2 h, there was an increase in CD16 expression on CD14^++^CD16^−^ subsets, while a further delay by 4 h at room temperature increased the number of CD16^+^ monocytes [40]. Thus, it is of note that variations in experimental protocols in these two studies can result in stark interpretation differences of the results.

As discussed earlier, monocyte subsets exist in a dynamic continuum in health and disease [36] and efforts to classify these subsets into discrete groups based on their surface antigens during disease progression may not be appropriate. Instead, it may be more instructive to consider the shift of the expression of CD14/CD16 along this continuum rather than the discrete frequencies of the subsets that can better predict disease prognosis [41]. A cross-sectional study involving 227 patients at high risk of cardiovascular disease supports this recommendation [41]. Greater mean fluorescence intensity of CD16 expression on the CD14^++^CD16^−^ subset, but not the absolute frequencies of the other subsets, predicted adverse cardiovascular events during follow-up.

## 5. Exercise Modulates Monocyte Subsets 

Regular physical activity and exercise training are associated with decreased inflammation and are related with a significant reduction in risk of cardiovascular-related mortality [13,42,43]. Although the mechanisms through which exercise exerts its cardio-protective effects are not fully understood, it may involve the modulation of the numbers or the functions of the monocyte subsets. Some of the exercise studies presented subsequently were published prior to 2010 and thus do not make distinctions between the CD14^+^CD16^++^ or CD14^++^CD16^+^ subsets. We have attempted as best as we can to describe these subsets given the limited amount of information available.

### 5.1. Effects of Acute Exercise

Addressing the impact of exercise on monocytes, Gabriel et al. [44] reported that four different types of anaerobic and aerobic exercises mobilize monocyte subsets to different degrees. These exercises comprised of (a) maximal running (anaerobic) for 1 min, (b) running for 24 min at 110% of individual anaerobic threshold (IAT), (c) running for 87 min at 100% of IAT, and (d) running 100 km in 8 h. Maximal running and running at 110% of IAT increased the relative proportion of CD14^+low^ monocytes (comprising the CD14^++^CD16^+^ and CD14^+^CD16^++^ subsets) to several-folds higher than baseline, compared with either running at 100% IAT or running 100 km, which did not result in a significant elevation of these subsets. 

Corroborating the findings of Gabriel et al., Steppich et al. [33] reported that a 1 min bout of cycle ergometry performed at 400 W (anaerobic) resulted in absolute numbers of CD14^+^CD16^+^ monocytes (comprising both the CD14^++^CD16^+^ and CD14^+^CD16^++^ subsets) increasing up to 2.1-fold after exercise, whereas comparatively, CD14^++^CD16^−^ monocytes were only increased by 1.3-fold. In agreement, Durrer et al. [45] found an increase in the number of circulating CD14^+^CD16^+^ monocytes immediately after a session of high intensity interval training (HIIT; 7 x 1-min intervals at 85% peak power output with 1-min rest periods at 15% peak power output between bouts). Interestingly, despite the increased numbers of CD14^+^CD16^+^ monocytes, this group reported a 18% decrease in CD14^+^CD16^+^ TLR-2 expression post-exercise, with values remaining 11% lower than pre-exercise values at 1-h post exercise [45]. Studies adopting longer-duration exercise protocols, have also demonstrated decrease in TLR-2 and TLR-4 expression post-exercise [46,47,48,49]. Radom-Aizik et al. [50] provided evidence of such exercise-induced reductions in TLR4 gene expression. 

Other studies also corroborate the aforementioned studies. LaVoy et al. [51] reported that maximal treadmill running (Bruce protocol) elicited a near 3-fold increase in circulating monocytes in 12 healthy and physically active men and women. In addition, the exercise protocol also resulted in 2- and 3-fold increases in the relative proportions of CD14^++^CD16^+^ and CD14^+^CD16^++^ monocytes, respectively. In comparison, there was a 20% decrease in the relative proportion of CD14^++^CD16^−^ monocytes in these subjects. Similarly, Slusher et al. [52] reported that maximal treadmill exercise performed by 25 healthy men resulted in a 7.7% decrease in the relative percentage of CD14^++^CD16^−^ monocytes, and 15.6% and 43.1% increases in relative percentages of CD14^++^CD16^+^ and CD14^+^CD16^++^ monocytes, respectively. Interestingly, there was decreased TLR4 expression on CD14^++^CD16^−^ and CD14^++^CD16^+^ monocytes but not in CD14^+^CD16^++^ monocytes. In another study, Booth demonstrated a 180% and 200% increase in CD14^+^CD16^++^ and CD14^++^CD16^+^, respectively, in eight trained cyclists immediately after a 60 km time trial [53]. Interestingly, CD14^++^CD16^+^ monocytes had a significant 21% increase in TLR2 immediately post-exercise and a 90% increase in TLR4 1-h post-exercise. 

It is worth mentioning that quantification of the monocyte subsets by LaVoy et al., were performed in vitro after isolated monocytes were incubated for 2 h at 37 °C. This differed with Slusher et al., and Booth et al., where flow cytometry was performed on identifying the monocyte subsets upon isolation from whole blood. Notwithstanding the differences in methodology, however, the results were similar for all three studies in that an acute bout of strenuous aerobic exercise increased the relative proportions of CD14^++^CD16^+^ and CD14^+^CD16^++^ monocytes, whereas the relative proportions of CD14^++^CD16^−^ decreased. 

In normotensive and hypertensive middle-aged participants (20–55 years), 20 min of treadmill running at 65–70% VO_2_ peak resulted in the increase in absolute counts of all three monocyte subsets [54]. However, when stratified for relative proportions, both CD14^++^CD16^+^ and CD14^+^CD16^++^ subsets increased, whereas CD14^++^CD16^−^ subsets decreased. A limitation in this study is the pooling of monocyte subset data from both hypertensive and normotensive individuals, which made interpretation of the results difficult. In agreement, Dimitrov et al. [55] demonstrated that in individuals with high prehypertension, the exercise-induced adrenergic increase in CD14^+^CD16^++^ monocytes in peripheral circulation was blunted compared to mild prehypertension and normotensive individuals. Hong and Mills [54] found post-exercise elevations in surface receptors of monocytes responsible for endothelial trafficking and adhesion (CD62L and CXCR2) to be greater in monocytes from hypertensive individuals, compared with normotensive individuals. While it is unclear whether the changes in such monocyte phenotypes are beneficial or detrimental for individuals with atherosclerosis, such findings indicate that exercise-induced mobilization and function of monocyte subsets vary within the clinical population. 

Exercise-induced catecholamine signaling via β_2_-adrenergic receptor has been shown to promote preferential mobilization of in CD14^+^CD16^++^ monocytes [56]. Interestingly, Rooney et al. [57] demonstrated that monocytes egress peripheral blood in only three minutes post 30 min of steady-state exercise; a higher rate than other leucocyte populations. Amongst the three subsets, CD14^+^CD16^++^ monocytes left circulation post-exercise at a quicker rate than the other two subsets. Gustafson et al. [58] reported a 2.7-fold increase in CD14^+^CD16^++^ monocytes compared to a 1.6-fold increase (*p* < 0.001) in CD14^++^CD16^−^ monocyte population following a maximal cycling test. As a non-significant increase was observed in the 2-h endurance protocol, the authors postulated (albeit not quantified) that the higher catecholamine release, cardiac output, lactate concentration, and more pronounced changes in pH could be factors affecting such mobilization. Matos et al. [59] observed a reduction in the relative percentage of CD14^+^CD16^+^ monocytes post one bout of aerobic exercise (three sets of 20 min at 60% VO_2_ max with 5 min passive rest) in obese insulin-resistant participants. 

As a major source of TNF-α [31], a reduction in the percentage of CD14^+^CD16^+^ monocytes could potentially reduce the progression of atherosclerotic lesions, as observed in a murine model (via reduction in pro-inflammatory markers and foam cells) [60,61,62]. TNF-α has specific roles in plaque formation and rupture as it directly influences macrophages, and endothelial and vascular smooth muscle cells (reviewed in Kleinbongard et al. [63]). TNF-α has been postulated to contribute to lipid and glucose metabolism [64], which, in a chronic setting, has direct implications for increased vascular risk [65,66]. Further, studies have demonstrated through administration of anti-TNF therapy, the role of TNF-α in endothelial dysfunction [67,68]. These studies demonstrated an increased flow-mediated dilation and reduced erythrocyte sedimentation rate, C-reactive protein, and disease activity score once the anti-TNF-α antibody neutralized its activities. Most recently, Dimitrov et al. [55] demonstrated that an exercise-induced rise in epinephrine concentrations decreased spontaneous and LPS-stimulated TNF-α production by monocytes. Such a finding supports the anti-inflammatory effects of exercises, even in a single 20 min bout of exercise (65–70% VO_2_ peak) [55]. 

Taken together, the results discussed above highlight that monocytic mobilization, function (e.g., cytokine production), and gene expression are dependent on the duration and intensity of exercise. Further, the timing of blood sampling is important, which as described earlier, can influence the expression of surface markers as well as the absolute quantity of individual monocyte subsets. Adrenergic response and increased expression of adhesion surface markers may promote tissue migration providing possible explanation for the different distribution of subsets in peripheral circulation reported post-exercise. Nonetheless, the studies support the anti-inflammatory effects of a single bout of exercise in both healthy and clinical populations.

### 5.2. Effects of Chronic Exercise Training

Regular exercise has been shown to exert anti-inflammatory effects and may mediate beneficial effects on atherosclerosis. In this section, we describe what is presently known about different types of exercise training on monocyte subsets and also peripheral blood mononuclear cells (PBMCs) in humans, given the dearth of research studies in long-term training and monocyte subsets per se.

In young sedentary men, 2 weeks of HIIT performed on a cycle ergometer three times each week increased the expression of TLR4 on CD14^++^CD16^−^ [69]. Training did not change the proportions of monocyte subsets, although there was increased TLR4 expression in most of the monocyte subsets (apart from CD14^++^CD16^+^). Concurrent aerobic and resistance exercise training may influence the circulating concentrations of the three monocyte subsets. In previously inactive elderly participants, combined endurance (20 min at 70–80% heart-rate reserve) and resistance exercise training (eight exercises, two sets at 70–80% of one repetition maximum) significantly down-regulated CD14^+^CD16^+^ (intermediate and non-classical) monocytes in the circulation by 64% [19]. In addition, there was a significant reduction of basal and LPS-stimulated TNF-α production [19]. It is difficult to directly compare monocyte subset responses to exercise in different populations (e.g., young vs. old) since the elderly population is more likely to demonstrate benefits from the anti-inflammatory effect of exercise, given that some degree of mild inflammation may already be present.

Few studies have investigated the effects of long-term exercise on monocyte subsets, although studies have reported beneficial effects in PBMCs or general inflammatory biomarkers. Gano et al. [70] demonstrated that two months of brisk walking (6 days/week, 50 min/day at 70% of maximal heart rate) by previously sedentary middle-aged and elderly adults significantly reduced gene expression of inflammatory biomarkers in PBMCs, such as the receptor for advanced glycation end products (RAGE), and monocyte chemotactic protein (MCP)-1. Furthermore, the long-term effects of exercise training on the atherogenic potential of PBMCs of 43 subjects (25 female; 18 male) at risk of developing ischemic heart disease were explored in a 6-month exercise intervention consisting of resistance and endurance activities (e.g., weight lifting and walking/running on a treadmill). The study also showed a down-regulation in the production of the inflammatory cytokines, such as IL-1α, TNF-α, and interferon (IFN)-γ, and an up-regulation of anti-inflammatory cytokines, including IL-4, IL-10, and transforming growth factor beta (TGF)-β via PBMCs at the end of the exercise program. It is unknown in this study how individual monocyte subsets contribute to the athero-protective phenotype. It is possible that alterations in the inflammatory milieu may result in the recruitment of different monocyte subsets.

A potential mechanism may implicate the increase in systemic vagal tone as an important signaling mechanism to spleen leucocyte populations such as T cells and monocytes. Narhrendorf and Swirski [71] suggest that such an increase in vagus nerve activity may reduce activation of inflammatory monocytes in the spleen, which may reduce the mobilization of such monocytes to the endothelium during the atherosclerotic process. 

## 6. Conclusions

There is preliminary evidence for the exercise-induced attenuation of inflammation [22], particularly by modulating the trafficking and circulating abundance and phenotypes (e.g., cytokine secretion in response to LPS) of different monocyte subsets [72]. Since inflammation is associated with the pathogenesis of atherosclerosis, such findings substantiate a role for exercise training in modulating the development of atherosclerosis. More clinical studies need to define the type (e.g., aerobic or resistance exercise) and amount of exercise training and their effects on these monocyte subsets. In particular, future studies need to elucidate the effects of exercise training on other molecular targets, including chemokine receptors and pattern recognition receptors. Furthermore, future research should clearly define the three monocyte subsets, since older studies usually did not make any distinction between the CD14^++^CD16^+^ or CD14^+^CD16^++^ subsets.
